# eHealth integration and interoperability issues: towards a solution through enterprise architecture

**DOI:** 10.1186/s13755-015-0009-7

**Published:** 2015-05-13

**Authors:** Olugbenga A Adenuga, Ray M Kekwaletswe, Alfred Coleman

**Affiliations:** Department of Information Systems, Witwatersrand, CLM Building #141 West Campus, 2050 Johannesburg, South Africa; School of Computing, University of South Africa, Florida Park, 1709 Johannesburg, South Africa

**Keywords:** eHealth, Enterprise architecture, Interoperability

## Abstract

Investments in healthcare information and communication technology (ICT) and health information systems (HIS) continue to increase. This is creating immense pressure on healthcare ICT and HIS to deliver and show significance in such investments in technology. It is discovered in this study that integration and interoperability contribute largely to this failure in ICT and HIS investment in healthcare, thus resulting in the need towards healthcare architecture for eHealth. This study proposes an eHealth architectural model that accommodates requirement based on healthcare need, system, implementer, and hardware requirements. The model is adaptable and examines the developer’s and user’s views that systems hold high hopes for their potential to change traditional organizational design, intelligence, and decision-making.

## Introduction

The recent industrial and economic meltdown due to workers’ demonstrations, threat from incurable diseases and, in addition, shortages in healthcare experts denies citizens in the rural community from benefiting from adequate medical care. These challenges force governments in developing countries to invest in information and communication technology (ICT) in healthcare. However, this investment failed to yield the intended purpose and therefore placed intensive pressure on the healthcare industry.

The researchers intend to focus on the enterprise architecture for successful implementation of ICT. The study progresses by first of all discussing the motivation for the study. Furthermore, the researchers also discuss various enterprise architectures in the enterprise architecture section and then proceed to propose eHealth architecture for healthcare.

## Motivation for the study

There is variation in the funding and expenditure in investment in eHealth from province to province in South Africa. In 2009, the annual budget for seven out of the nine South African provinces were as follows: Gauteng spent South African Rand 188.3 million (approximately US$ 18.83 million); Limpopo, South African Rand 178.6 (approximately US$ 17.86 million); KwaZulu-Natal, South African Rand 105 million (approximately US$ 10.5 million); Free State, South African Rand 32 million (approximately US$ 3.2 million); Northern Cape, South African Rand 20.4 million (approximately US$ 2.04 million) and North West’s spending was South African Rand 15 million (approximately US$ 1.5 million). The Bill and Melinda Gates Foundation (BMGF) categorise eHealth for system as scope, scale, and sophistication. This category is in five stages. The stages are as follows:

Stage 1, paper-based systems for collecting district health indicators; Stage 2, optimisation of paper systems through simplifying indicators and reducing duplication; Stage 3, migration of traditional district health information systems to electronic storage and reporting; Stage 4, introduction of operational ICT systems as a source of data for HIS; and Stage 5, a fully comprehensive and integrated national HIS. South Africa’s healthcare system is placed at stage 3; this forces the country to, therefore, propose an eHealth strategy between 2012 and 2016 in order to move to stages 4 and 5. Based on the foregoing premise, the National Department of Health (NDoH) recognised the need to: (a) Implement patient-based information systems at all facilities where healthcare is delivered. (b) Link all these systems to a national electronic health record system. (c) Derive all indicator data from patient data captured electronically at the point of care. (d) Establish a unique identifier for each South African. (e) Ensure effective registration of births and deaths. (f) Support access from all facilities to all records in other facilities.

To achieve the eHealth strategy, there is a need to have an eHealth architecture for the eHealth strategy in the country [1]. The need for research effort comes from the fact that large sums of money have been used to procure health ICT and HIS in South Africa in the past. Nonetheless, the ICT and HIS within the health system are not meeting the requirements to support the processes in operation within the healthcare system. This renders the healthcare system incapable of adequately producing data and information for management and for monitoring and evaluating the performance of the national health system. Illustration given by [2] in the editorial comment for eHealth International stated ‘that hardware and software applications that have been implemented in hospitals, trusts, or country-wide have ended up wasting thousands and often billions of dollars and have become obsolete before long because of a lack of open discussion about their appropriate use’.

The problems with the investment and failures to achieve the outcome of ICT and HIS implementation in the health system are highlighted below: (a) A large number of disparate systems between which there is little or no interoperability and communication. (b) Silos of information within levels of government, government departments and programmes within the national and provincial departments of health, resulting in duplication of effort and disparities in reporting. (c) The need for strong information governance to ensure compliance with the necessary standards and procedures for, and appropriate use of, health information (both patient-based and aggregate).

The aim of this study is to propose architecture for eHealth, which could enhance eHealth success within the country. The objective of this study is to propose eHealth architectural model for healthcare delivery for developing countries.

## Enterprise architecture

ISO 15704 described architecture as the basic arrangement and connectivity of parts of a system. TOGAF [3] describes architecture as various means, depending on its contextual usage: a formal description of a system at the component level to guide its implementation; the structure of components, their interrelationships and the principles and guidelines governing their design and evolution over time; and organisational structure of a system or component.

Enterprise architecture (EA) is a means of a high level of abstraction of communication with and among stakeholders. It represents stakeholders’ anticipation of attributes of enterprise system rather than documenting detailed requirements on functions, data or resources that will be specified in the later stage of enterprise system implementation.

The responsibility of the architect is to deal with concerns, show how these concerns and the requirements are going to be addressed, and document the transactions that are going to be made in reconciling the potentially conflicting concerns of different stakeholders. Without the architecture, it is highly unlikely that all the concerns and requirements will be considered and met [3]. Normally, enterprise architecture ought to show properties that can be verified with regard to user needs, such as open or closed architecture, interoperable or compatible, and centralised or decentralised.

Therefore, an EA should be organised in a manner that supports any reasoning about the structure, properties and behaviour of the system. EA identifies various parts that make up the overall system and provides a plan from which the system can be developed. EA also allows the managing of complexities due to various factors, such as technology, size, interface, context and stakeholder’s need. It aims at creating an ideal future system; this idea is represented as a high abstraction level solution that lays down the foundation for a design. It is the blueprint that focuses on the essential features and characteristics of the system.

## Enterprise integration

Enterprise integration is the modus operandi used in ensuring the interaction between enterprise entities, which is necessary in achieving domain-specific objectives. Enterprise integration can be approached in various ways and at various levels [4]. The approaches are physical integration, which involves interconnection of devices, connecting machines via computer networks; application integration, such as integration of software applications and database systems; and business integration involving coordination of functions that manage, control and monitor business processes. Some other approaches also consider integration through enterprise modelling, for example, through the use of a consistent modelling framework [5] and integration as a methodological approach in order to achieve consistent enterprise-wide decision-making [6].

## Enterprise interoperability

Enterprise interoperability is the ability for two systems to understand each other and to use the functionality of each other. The word “interoperate” implies that one system performs an operation for another system [7]. From a computer technology point of view, it is the faculty for two heterogeneous computer systems to function jointly and to give access to their resources in a reciprocal way [7]. In the context of networked enterprises, interoperability refers to the ability of interactions, exchange of information, and services between enterprise systems. Interoperability is considered as significant if the interactions can take place at least on three different levels: data, services and processes, with semantics defined in a given business context. Interoperability has the meaning of coexistence, in general, autonomy and federated environment, whereas integration refers more to the concepts of coordination and coherence.

## eHealth architecture for healthcare

The challenge with the absence of architecture arises as a result of globalisation, competition, and fear of survival, taking into account advancements in information systems. This has led to more efficient and effective ways in using information systems within medical operations. To accomplish the eHealth architecture and ultimately improve operation processes for healthcare, the researchers will study the interoperability of information systems enterprise architecture model to achieve the objectives in the healthcare environment. The adoption of appropriate enterprise architecture and the principle of management of information systems will eliminate the eHealth interoperability challenge [8, 9]. As pointed out in [10] architecture will provide an extensive impact on the operation of healthcare delivery. He added that the architecture will define the structure of healthcare, which will dictate the capabilities of healthcare in using eHealth.

With regard to Zachman’s model for information systems architecture, introduced in 1987, other enterprise architecture models could be the solution to the interoperability problem in eHealth [8]. Some of the enterprise architecture models are the open group architecture model, the department of defence architecture model, and the federal enterprise architecture model [4, 11]. The inadequacies of the existing models are as stated: the existing inflexibility of the requirements of IT in relation to business change; the incapability of multiple approach usage in the existing models; and the existing boundary prevents future IT requirements from meetings business needs. All the architectural models, according to [12], differ in content and target audience. The open group architecture model details the process of creating architecture with less emphasis on actual modelling, while the department of defence architecture model’s emphasis is on models and metamodels. All of the existing architecture compromises on the content of architectural models of interoperability.

An eHealth architecture framework based on the shortcoming of the previous enterprise architectural model will be proposed. First, the eHealth architecture considers the implementation requirement beyond what is required at present. Secondly, the eHealth architecture framework will accommodate requirements and any IT projects implementation requirements for the healthcare. Thirdly, for the purpose of an academic environment, the contribution will be a step towards an idea which can be a baseline for achieving a unified model of architecture for eHealth. If the eHealth architecture is fully adopted, the healthcare industry stands to receive many benefits, such as reduction of cycle time; faster response to patients; better financial management; it could be an outline for e-commerce; link organisations’ function seamlessly; and make tacit knowledge explicit within the healthcare industry [13–15].

## Enterprise architecture models

The prominent work that is related to identifying components of enterprise or eHealth architecture is the enterprise architecture model, which is based on a common information technology domain [4] for improving interoperability among heterogeneous information systems. The architecture was created to deal with the weaknesses on the Zachman model, the open group architecture model, federal enterprise architecture model, and enterprise architecture model for a common information technology [4].

### Zachman architecture model

John Zachman presented an enterprise architecture model in 1987, which consists of six columns, various views, and five rows. The Zachman enterprise architecture is today recognised as ontology. The model presents the perspectives of planner, owner, designer, builder, and sub-contractor [8, 16, 17]. The attribute of this model is the 5W1H [15], which stands to question the 5Ws and H, i.e. what, where, who, when, why, and how? The model advantage is that it provides clarity to a complex enterprise. It is, in addition, a model that describes enterprise business requirements in information technology. The disadvantages come from the fact that there is no procedure in the application of the architectural model. Also, the model is too idealistic, which makes it difficult to define a product base on this model.

### Federal enterprise architecture model

The federal enterprise architecture model was introduced in 1998 by the Chief Information Office (CIO) consortium. This architecture provides guidance for enterprise integration information technology to the United States government. The architecture model prioritises certain architectural segments, while it also provides the mechanism for identification, development, and documentation [4, 8]. In addition, the architecture advantage is that it standardises the organisation’s mission and vision, which makes it better in enhancing effectiveness. The drawback of this model is that it has no template or products for development.

### The open group model

The open group model, developed by the Architecture Forum in the mid-1990s, with its first version presented in 1995, was based on the technical architecture model for information management. This architecture provides a comprehensive approach for designing, planning, implementing and governing enterprise information architecture [18, 19]. It has a holistic approach to design, modelled at business, application, data, and technology; however, it depends on modularisation, standardisation and already-existing technologies.

### Department of defence architecture model

The department of defence architecture model’s first version was developed in the 1990s as C4ISR (Command, Control, Communications, Computers, and Intelligence, Surveillance, and Reconnaissance) architecture model. This model can be classified as a descriptive model, according to [20], which acts as a mechanism for visualising, understanding, and assimilating the scope and complexities of architecture. On the other hand, it is only suitable for large-scale systems. It specifically details an external operating domain for customers to operate.

## eHealth architecture for healthcare

Based on Zachman’s model [21, 22], eHealth architecture provides taxonomy for the insight that describes the real-world ICT and HIS implementation. This section introduces the eHealth architecture for healthcare in a developing country. The model provides the solution as a result of the weaknesses in the existing architectural models. The model relies on the Zachman model, on the grounds that it is ontology for enterprise architecture model modelling [23, 24]. The thought of adopting the Zachman model is as elaborated in [20] as the dynamic and uncertain healthcare environment. Therefore, the following are the reasons for the adoption; the process of modelling social–technical systems or ontology such as a unified architecture model helps in identifying and understanding the relevant elements in a specific domain and the relationships between them [23, 24] the use of s formalised Zachman enterprise architecture model (i.e. ontology) helps managers to communicate easily and share their understanding of e-business with other stakeholders [25] and mapping and using enterprise architecture as a foundation for discussion that facilitates change. Healthcare activities in using ICT can easily be modified for certain elements of its architecture model. The eHealth architecture will help in identifying the relevant measures to be relied on in the change process within the healthcare [26]. The eHealth architecture will help managers simulate rules, change managements and capabilities to learn about them. This is a way of executing risk-free experiments without endangering delicate healthcare activities [27]. The eHealth architecture is in the form of matrix modelled architecture, with an n-by-m matrix, which comprises n rows, which represent perspectives and m columns, which represent views. The perspective in this context comprises the enterprise, system, implementer and the hardware [21, 22] and the views are data, function, organisation, and infrastructure. The requirement entries in the row are represented by x rows, and the entries for the column are represented by y columns, which is denoted by

$$\begin{aligned}&a_{ij}\ \mathrm{The\, relationship\, between\, and\, the\, matrix\, shown\, below\, are}\\&a11, a12, \ldots , a1m\ \mathrm{represents\, the\, horizontal\, integration\, and}\\&a_{11}, a_{21}, \ldots , a_{n1}\ \mathrm{represents\, the\, vertical\, integration\, of\, enterprise\, requirements\, [4]}. \end{aligned}$$

The matrix shows the extent to which healthcare requirements can be constructed in order to realise the goals of ICT and HIS in healthcare.

Assuming that the horizontal integration of

$$\begin{aligned}&a_{11}\ \mathrm{represents\, data\, within\, the\, healthcare,\, then,\, in\, its\, row,\, there\, will\, be}\\&x_{1},x_{2},\ldots ,x_{n}\\&a_{12}\ \mathrm{represents\, a\, function\, within\, the\, healthcare,\, then,\, there\, will\, be\, in\, its\, row}\\&x_{1},x_{2},\ldots ,x_{n}\\&a_{13}\ \mathrm{represents\, organisation\, within\, the\, healthcare,\, then,\, there\, will\, be\, in\, its\, row}\\&x_{1},x_{2},\ldots ,x_{n}\\&a_{1m}\ \mathrm{represents\, infrastructure\, within\, the\, healthcare,\, then,\, there\, will\, be\, in\, its\, row}\\&x_{1},x_{2},\ldots ,x_{n} \end{aligned}$$

Also, if, the vertical integration is represented as

$$\begin{aligned}&a_{11}\ \mathrm{represents\, different\, departments\, within\, the\, healthcare,\, then,\, there\, will\, be\, in\, its\, row}\\&y_{1},y_{2},\ldots ,y_{n}\\&a_{21}\ \mathrm{represents\, different\, systems\, within\, the\, healthcare,\, then,\, there\, will\, be\, in\, its\ row}\\&y_{1},y_{2},\ldots ,y_{n}\\&a_{31}\ \mathrm{represents\, data\, within\, the\, healthcare,\, then,\, there\, will\, be\, in\, its\, row}\\&y1,y2,\ldots ,yn\\&a_{n1}\ \mathrm{represents\, data\, within\, the\, healthcare,\, then,\, there\, will\, be\, in\, its\, row}\\&y_{1},y_{2},\ldots ,y_{n} \end{aligned}$$

The eHealth architecture enables various healthcare elements to comprehend the detailed structure and components of the healthcare and how they work together. [12] asserts the common values of the eHealth architecture, which are as follows: readily available reports of various functions within the healthcare; the ability to unify and integrate the healthcare processes across multiple functions; ability to unify and integrate data across the healthcare and to link up with external partners; increased agility by lowering the complexity barrier within the healthcare; reduced healthcare solution delivery time and development costs by maximising reuse of the healthcare model; and ability to create and maintain a common vision of the future shared by both healthcare and IT communities by driving IT alignment. Combining the horizontal and the vertical integration from the equation above, it follows that, for Enterprise architecture, there will be

$$\begin{aligned}&x_{i}y_{j} = x_{1}y_{1} + x_{2}y_{2} + \cdots + x_{n}y_{m}\\&x_{i}y{j} = x_{n}y_{m} + x_{2n}y_{2m} + x_{2(n-1)}y_{2(m-1)} \end{aligned}$$

System architecture, gives

$$\begin{aligned}&x_{i}y_{j} = x_{1}y_{1} + x_{2}y_{2} + \cdots + x_{n}y_{m}\\&\cdots\\&x_{i}y_{j} = x_{n}y_{m} + x_{2n}y_{2m} + x_{2(n-1)}y_{2(m-1)} \end{aligned}$$

Implementation architecture, gives

$$\begin{aligned}&x_{i}y_{j} = x_{1}y_{1} + x_{2}y_{2} + \cdots + x_{n}y_{m}\\&\cdots\\&x_{i}y_{j} = x_{n}y_{m} + x_{2n}y_{2m} + x_{2(n-1)}y_{2(m-1)} \end{aligned}$$

Hardware architecture, gives

$$\begin{aligned}x_{i}y_{j} = x_{1}y_{1} + x_{2}y_{2} + \cdots + x_{n}y_{m}\end{aligned}$$

The architectural framework being applied towards a solution through enterprise architecture in eHealth integration and interoperability is adapted from [28], irrespective of the way the horizontal and vertical integration of eHealth as explained in the equations above. The eHealth model presents the requirement for present and future ICT and HIS in healthcare; the model examines requirements based on the healthcare need, system, implementer, and hardware requirements. The model is adaptable and examines the developer’s and user’s views that systems hold high hopes for their potential to change traditional organisational design, intelligence, and decision-making for the better, according to [29].

The eHealth architectural model also defines dynamic capability as the healthcare’s ability to integrate, build, and reconfigure internal and external competences to address rapidly changing environments. Changes in technology bring about new possibilities in the business environments. Since information technology technical solutions are not static, organisations are faced with continual change hardware, software, and networking standards.

$$\begin{aligned}x_{i}y_{j} = x_{n}y_{m} + x_{2n}y_{2m} + x_{2(n-1)}y_{2(m-1)}\end{aligned}$$

As new software becomes available, hardware must be replaced to meet the minimum requirement of the software [27]. As this phase of change occurs in technology, firm’s innovation performance, aspiration level, and organisational learning, according to [30] that in order to be innovative, an organisation should develop its absorptive capacity. The change in technology forces a shift in the scope of technology implementation; thus, the unified enterprise architecture should create an avenue for this change to be accommodated [31].

$$\begin{aligned}&x_{i}y_{j} = K\sum ^{m}_{i=1}[x_{i}y_{j}.\ln (x_{i}y_{j})]\,(i = 1,2,\ldots,n)(j = 1,2,\ldots,m)\\&K = \infty /\ln (m)\\&x_{i} = y_{j}\bigg/\sum ^{n}_{i=1}y_{j}\,(i = 1,2,\ldots,n) \\&y_{j} = x_{i}\bigg/\sum ^{m}_{j=1}x_{j}\,(j = 1,2,\ldots,m) \end{aligned}$$

## Discussion

This paper presents the eHealth architecture. The purpose of the study is to only use matrix with numbers as entries that should be considered before embarking on the implementation of eHealth. The entries in the matrix can be a function in the requirements to have a successfully implemented eHealth technology. This should be organised for the integration of healthcare and IT management based on the vertical and horizontal integration and interoperability between information systems. The relationship between the matrix shows that

$$\begin{aligned}a_{11}(t), a_{12}(t), \ldots , a_{1n}(t)\end{aligned}$$

represents the horizontal integration, and

$$\begin{aligned}a_{11}(t), a_{21}(t), \ldots, a_{m1}(t)\end{aligned}$$

represents the vertical integration of enterprise requirements change with time. Similarly, [32] the vertical and horizontal integration for the eHealth adoption is discussed in the context of organisational, learning, and ease of use of the technology.

### Barriers in the organisational context in healthcare

Barriers to the implementation of eHealth technology appear to be the high initial costs and uncertain return on investment; these barriers discourage the healthcare sector from being unwilling to implement the technology. Physicians that lack financial support, with the costs required to implement eHealth technology, were highly less likely to have implemented the technology. In addition, changes in practice patterns brought about by the implementation of electronic medical records also constitute a barrier to adoption of eHealth technology. The time and effort involved in learning to use these technologies is another significant barrier. The lack of a strategic plan for implementing the applications and difficulty in recruiting experienced IT personnel to manage the eHealth technology is another major barrier. Inadequate design during implementation of eHealth and difficulties in entering progress notes require physicians to spend extra time entering patient information. Exchanging clinical data between laboratories and hospitals has proved to be a hindrance to the successful implementation of eHealth. Privacy concerns of eHealth raise another barrier as this technology will rely on the Internet, and patients’ information could be intercepted by unauthorised individuals. Both physicians and the public are concerned about potential breaches of confidentiality. Since many eHealth systems rely on web-based applications, many physicians and patients fear that medical records may not be secure.

### Barriers to learning

Learning is a factor that can facilitate participation and allow healthcare experts in harnessing the full potential of eHealth technology. A barrier to learning can result from ageing and its effect on learning and memory. Boulton-Lewis et al. [33] reports that cognitive tests to access information processing speed, working memory capability, and declarative learning, administered on adults aged 17–86 years old found significant age-related decrements in relation to ages. On the other hand, knowledge already acquired has the ability to be retrieved by individuals, and proper visual and auditory sensory capabilities are maintained for the most part, especially among individuals who are in their 1960s [33]. Motivation is another factor that enables healthcare experts to be able to attain the same learning outcomes as younger learners. It is also evident that the nature and amount of learning achieved in early and middle life impacts significantly on the competence of older adults when learning new skills and acquiring new knowledge. Motivation and confidence are also critical to learning at any age, and particularly so as people become older [34]. Ziefle et al. [35] emphasises that older adults learn more slowly and need more practice, but in most cases, their motivation will be strong enough to enable them to learn new skills. They also declared that significant factors for learning activities were good physical health, level of prior education, good mental or emotional health, being younger, living in regional areas, not being retired, and being a high-income earner.

### Barriers result from ease of use in eHealth

eHealth technology has the potential to change the way health services are delivered to patients and to improve the quality of health services by providing easy access to healthcare in developing countries, especially in sub-Saharan Africa. The benefits of eHealth as a technique to augment the quality of healthcare are acknowledged worldwide, and healthcare institutions are determined to be acquainted with the way of delivering their services efficiently and effectively. The benefits of telemedicine have been summarised as enhanced access to information, provision of new healthcare services, improved access to existing service, increase in care delivery, improved professional knowledge, better quality control of screening programmes, and reduction in healthcare costs. The effective use of eHealth can help surmount geographic remoteness for the populations that live in remote areas, as it provides easy access, dissemination, utilisation, and exchange of information on combating diseases such as malaria, tuberculosis and HIV/AIDS [36]. Demski et al. [37] identified similar benefits, which include the use of eHealth to facilitate communication between different levels of delivery units (e.g. district hospitals, health centres, clinics and referral hospitals). At the referral level, there is added significance through effective resource management and planning, efficiency in processing transactions, and access to more reliable information. Healthcare professionals would have the advantage of effectively and efficiently sharing the information with other professionals [36]. ICT such as email, telephone or mobile technologies facilitate communication between two or more professionals by sharing information on various diseases that may afflict the patients. The use of eHealth in healthcare has the potential to improve access to educational opportunities for professionals and access to care in remote areas. The use of eHealth may not deliver the expected health benefits automatically. Before the application of eHealth in health can become a real success, numerous challenges that currently serve as obstacles to their effective utilisation need to be resolved. Health stakeholders may be unwilling to use certain eHealth due to certain beliefs, or they may be resistant to change due to unfamiliar ICT (e.g. telephones, computers). Resistance to change can, therefore, hinder eHealth use in health institutions; for example, to record patient data in manual files, they might be resistant to using a computer for the first time if they have not been given proper training or administration support.

## Conclusion

This paper explored an enterprise architecture framework with the view of proposing an eHealth architectural model whose significance is to address eHealth integration and interoperability issues. Changes in technology bring about new possibilities in the healthcare environments. The effort of the researchers shows that information technology technical solutions are not static; organisations such as healthcare institutions are faced with continual change in hardware, software, and networking standards. As new software becomes available, hardware must be replaced to meet the minimum requirement of the new software. The future of technology development may be unknown though, but it is enough to say that technology will continue to evolve at a fast pace. Therefore, the healthcare industry should anticipate future demands, which will result from technological change. In this aspect, the architecture to be relied on by the healthcare industry should have the capability to meet current demands while also maintaining the capability to meet anticipated future demands. Furthermore, healthcare industries have to realise the importance of architecture before considering an IT solution to serve their needs. However, it is too early for eHealth architecture to be used as a basis for healthcare, ICT and HIS. This research will continue, and in the future, data will be used to validate the outcome of the study. The analysis used in this paper is descriptive, and full data analysis regarding the integration and interoperability issues generated by the applicability of eHealth usage will be analysed in future research.
